# Optimizing the Mechanical Properties of Cement Composite Boards Reinforced with Cellulose Pulp and Bamboo Fibers for Building Applications in Low-Cost Housing Estates

**DOI:** 10.3390/ma17030646

**Published:** 2024-01-29

**Authors:** Anuoluwapo S. Taiwo, David S. Ayre, Morteza Khorami, Sameer S. Rahatekar

**Affiliations:** 1 Composite and Advanced Materials Centre, Cranfield University, Cranfield MK43 0AL, UK; samuel.taiwo@cranfield.ac.uk (A.S.T.); d.s.ayre@cranfield.ac.uk (D.S.A.); s.s.rahatekar@cranfield.ac.uk (S.S.R.); 2 Department of Metallurgical and Materials Engineering, Federal University of Technology, Akure PMB 704, Ondo State, Nigeria; 3 Faculty of Engineering, Environment and Computing, Civil Engineering Department, Coventry University, Coventry CV1 2TU, UK

**Keywords:** natural fiber, kraft pulp, Hatschek process, non-asbestos cement board, construction materials

## Abstract

Africa is the third-richest continent in the world in terms of bamboo species. Despite these laudable natural resources, most African countries still use asbestos cement board as one of their major building materials. This is chiefly due to the high cost of equipment and technologies associated with non-asbestos-fiber cement board production. The current research seeks to underscore the possibility of utilizing these massive continent resources for non-asbestos-fiber cement board production by employing the existing production process in the asbestos cement industries via an innovatively developed laboratory-simulated Hatschek process. Non-asbestos-fiber cement boards incorporating kraft and bamboo fibers were successfully produced in the laboratory using this innovative method based on Hatschek technology, with natural fibre addition in the range of 2–6 wt.%. Experimental results revealed that the Flexural strength and deflection of the board improved significantly, producing optimum values of 10.41 MPa and 2.0 mm, respectively for composite board reinforced with 10 wt.% and 6 wt.% of kraft pulp and bamboo fibers, respectively. The SEM morphology of the fractured surfaces revealed the mode of composite fracture as well as good interaction at the fiber–matrix interface. Overall, the mechanical properties of the developed composite boards satisfy the minimum requirements of relevant standards based on fiber cement flat sheets and can be employed for internal building applications in low-cost housing estates in developing countries. The outcome of this research indicates that the current industrial production process based on Hatschek technology can be employed for non-asbestos-fiber cement board production using the studied natural fiber.

## 1. Introduction

Since the early 80s, there has been a continuous discussion on using vegetable fibers to create low-cost, sustainable fiber cement composite sheets in many parts of the world [1,2,3,4]. This has been due to the never-ending health concerns [5,6] associated with asbestos-fiber-reinforced ceilings and roofing that were used in building constructions before this time. Through the adaptation of a highly expensive production process, many developed nations of the world have been able to successfully produce asbestos-free cement composite boards utilizing cellulose fibers obtained from hardwoods or softwoods for building applications [7]. However, many developing countries cannot access this technology due to its monopoly and the high cost of equipment and machinery involved. Hence, they have continued the utilization of asbestos-fiber-based cement boards. Thives et al. [8], in their attempt to answer the notorious question ‘Is asbestos still a problem in the world’, reported that asbestos cement still makes up around 74% of the 190 million m^2^ of fiber cement composites made each year in Central, Southern, and Western Africa, primarily as corrugated roofing components. Chrysotile asbestos is being banned in a few African nations (South Africa, for example) due to the continuous growing health concerns [6,8]. However, according to some recent research [9,10,11] and review reports [5,6,8], asbestos mining, production and use continue in many developing countries, particularly those on the African continent. Therefore, new products that meet consumer demands in each application area must be developed using existing and/or new raw materials and production techniques. Several investigations on the application of cellulose fibers in combination with polymeric fibers, agricultural waste fibers, and glass fibers have been conducted by many researchers in this regard [10,12,13].

The cultivation and production of plant fiber in the Sub-Saharan African regions is a well-known venture [14], as is the ensuing creation of by-products from commercial, agricultural, and industrial operations. The resultant cellulose fibers offer enormous potential for fiber cement manufacturing at a much lower cost than those associated with the use of typical kraft wood pulps produced for the paper industry because of the very low cost of raw materials and simplified production procedures based on existing technologies. Natural fibers present significant benefits such as their low density, suitable mechanical and stiffness characteristics, high disposability, and renewability [15,16,17]. They are also biodegradable and recyclable [18,19]. All these characteristics of natural fibers have made their use as reinforcements become a subject of extensive investigation. For instance, Hasan et al. [20] and Gupta et al. [21] extensively discussed the potential of natural fibers as promising reinforcing materials for various engineering applications. In another report by Khorami and Ganjian [22], an emphasis was laid on the use of by-products obtained from agricultural wastes for several engineering and industrial applications. All the evidence from this research points to the fact that natural fibers, at some point in the future, may outsmart synthetic or man-made fibers in many areas of applications.

Africa is the third-richest continent in the world for bamboo species after South America and Asia [23,24]. Most of Sub-Saharan Africa, from Ethiopia to Madagascar and South Africa, is covered in bamboo. Several temperate bamboo species, including Bergbambos, Oldeania, Thamnocalamus, and Yushania, as well as at least four genera of tropical clumping bamboos, including Cathariostachys, Cephalostachyum, Oxytenanthera, and Schizostachyum, are found on the continent [24,25,26]. In Africa, several types of bamboo are also grown for economic purposes [23]. With over 1.47 million hectares of bamboo cover, Ethiopia is recognized as having the most bamboo cover of any African nation [24]. Bamboo is an extremely resilient crop that can be used as an inexpensive, sustainable substitute for building materials in underdeveloped and developing nations. Since bamboo fiber is readily available, inexpensive, and rapidly renewable, it is one of the natural fibers that can be effectively employed as reinforcing materials. As a result, many researchers have attempted to incorporate bamboo into cementitious composites [27,28,29,30,31,32,33]. Furthermore, because of its high strength-to-weight ratio, bamboo fibers can significantly increase the flexural strength and fracture toughness of cementitious-based composites while also lowering the laminate’s overall weight [32].

Hence, the current research objectives are to evaluate the potential of bamboo fiber as a reinforcing element in fiber cement boards (FCBs) produced using a specially developed method based on the Hatschek process. This is because bamboo is a valuable natural fiber with abundant availability on the African continent. The report includes a clear assessment of the mechanical, microstructural, and durability performance of composite boards made from individual fiber incorporation.

## 2. Materials and Methods

### 2.1. Materials

The World of Wool Company, a trading name of Europa Wools Ltd., Huddersfield, UK, provided the industrially processed (hydrolysis/alkalization and bleaching of raw plant stems) raw bamboo fiber used in this study. The primary source of kraft pulp fiber was recycled cardboard (cartons) paper. High-strength (HS52) BSEN 197-1 BSEN I, 52.5 N Ordinary Portland Cement was provided by Hanson Heidelberg Cement Group, Maidenhead, UK. Other materials such as the Conical flask capable of measuring 1000 mL, Edwards high-vacuum pump with model no. EDM 2 having a speed of 1425 rpm, rectangular steel mold measuring 180 × 80 mm, rubber bung, hose, and control valves needed to replicate the Hatschek process were provided internally by the Composites and Advanced Materials Centre, School of Aerospace, Transport, and Manufacturing at Cranfield University, United Kingdom.

### 2.2. Methods

#### 2.2.1. Bamboo Fiber Preparation

**Figure 1:**
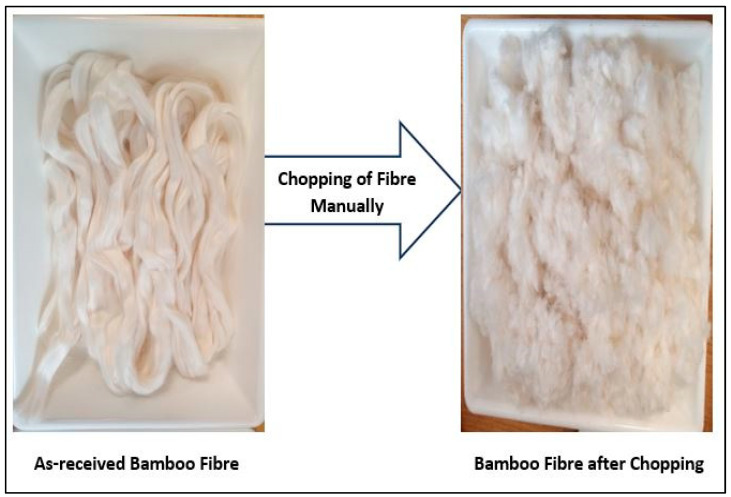
Camera photo of the as-received raw bamboo fiber and the prepared chopped fiber.

The raw bamboo fiber provided by Europa Wools Ltd. is a long-strand, continuous fiber with an approximate length of 4 m per 100 g of fiber. The as-received fibers cannot be used in their present form; hence, the fiber was chopped manually into short and discrete fibers with a length ranging from 4–6 mm before incorporation into the cement boards. Figure 1 shows a camera photograph of the bamboo fiber used in this research and its preparation process.

#### 2.2.2. Characteristics of Bamboo Fiber

The physical and mechanical properties of the bamboo fiber used in this current study are presented in Table 1; Tensile strength and Young’s modulus were conducted as per ASTM D3822-07 [34] guidelines using the DEBEN micro tester MT200 single-leadscrew Tensile Tester sourced from Deben UK Limited, Suffolk, United Kingdom and equipped with a Leica S9D 0.5× microscope sourced from Switzerland Ltd. Singapore and a soft cardboard mounting card with a gauge length of 10.2 mm over a 5 N load cell at a motor speed of 0.1 mm/min. In each instance, ten (10) samples of the single fibers were examined, and the average results are presented in Table 1.

#### 2.2.3. Thermogravimetric Analysis (TGA) of Bamboo Fiber

To evaluate the decomposition and stability of the bamboo fiber in the alkaline media of the cement matrix, TGA was conducted. Using the ramp method and a TGA Q500 TA instrument in a nitrogen atmosphere, the thermal properties of bamboo fiber were recorded over a temperature range of 30 to 600 °C at a heating rate of 10 °C/min.

#### 2.2.4. Kraft Pulp Fiber Production

**Figure 2:**
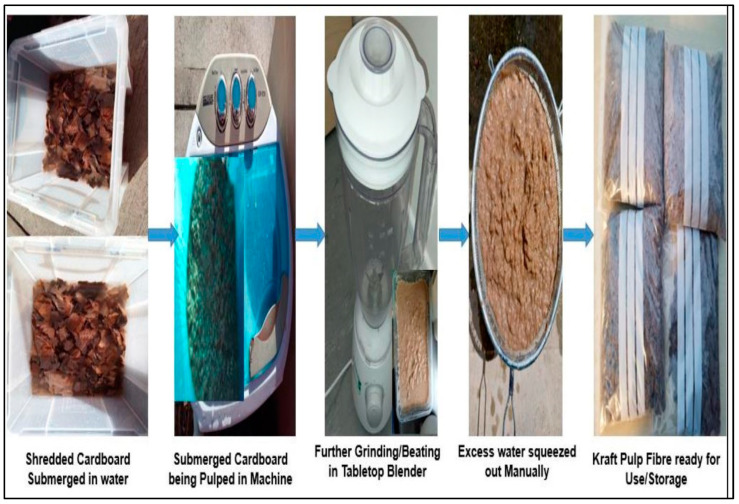
Pulping process for producing kraft pulp fiber.

The primary source of kraft pulp fiber was recycled cardboard (cartons) papers, which were sourced from paper shops on the Cranfield campus. The waste cardboard paper was first cut into smaller pieces by hand. The cardboard papers were then soaked in potable water for 48 h, with a cardboard-to-water ratio of 1:5 by weight of the cardboard. Next, the cardboard was pulped using a GWL-82718 LEISURE DIRECT mini washing machine sourced from UK Leisure Direct Ltd., Erdington, United Kingdom, for 2 h, and finally, it was ground at a low speed in a Kitchen Table-top Blender with model TBBL20 for an additional 8 to 10 min. The water used for the pulping process was manually extracted from the product received from the blender. The final product was kept in zip bags and refrigerated at 3 ± 1 °C. The weight of oven-dried kraft pulp fiber divided by the weight of fiber plus water yielded an average moisture content of 70% for the kraft pulp fiber. A pictorial representation of the kraft pulp fiber production process is shown in Figure 2.

#### 2.2.5. Production of Fiber Cement Composite Boards

The Hatschek method invented by Ludwig Hatschek in the 1890s was the first production process used industrially for the production of fiber cement boards. Ludwig used a combination of cellulose, reinforcing fibers, and Portland cement in water to form a slurry, which was fed into a typical paper-making machine in which a cylindrical sieve or sieves rotated through the slurry and then via a conveyor belt to form a fiber cement flat sheet [12]. This Hatschek method was innovatively simulated in the laboratory and used as the production method for the fiber cement board in this study.

Using the laboratory-replicated Hatschek process, fiber-reinforced cement composite boards with different proportions (2, 4, and 6 wt.%) of bamboo fibers were manufactured in the lab using the bamboo fiber as individual reinforcing fibers. Following the mix design shown in Table 2, the individual materials (kraft pulp, bamboo fiber, and cement matrix) were weighed using a Scout Pro digital weighing balance with a 6-digit display and an accuracy of ±0.01 g.

**Figure 3:**
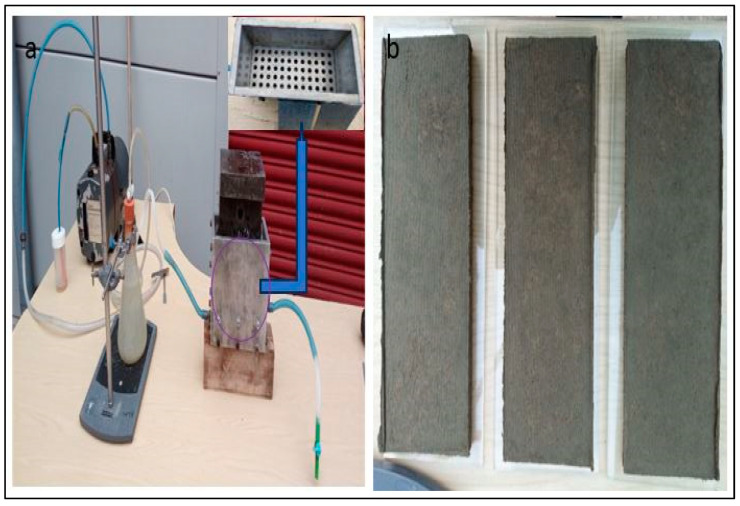
(**a**) Setup for the lab-simulated Hatschek process. (**b**) A selection of manufactured specimens in the lab.

To produce fiber cement composite boards, a known weight of kraft pulp fiber was first mixed with 750 mL of water for about 5 min using a portable handheld mixer operating at a speed of 600–1900 rpm. This is a way of ensuring that the fibers from the kraft pulp are dispersed evenly throughout the water. Subsequently, a suitable quantity of bamboo fiber and the cement matrix was added to the kraft pulp mixture, and all the constituents were thoroughly stirred for an additional 5 min to attain homogeneity. Following mixing, the resultant slurry was poured into the pre-assembled 180 mm by 80 mm rectangular steel mold in sequential layers, as depicted in the mold setup in Figure 3a. After turning on the vacuum pump attached to the mold, the extra water in the combined slurry was drawn out of the mold. One end of the mold was attached to a conical flask, which was used to collect the extra water. With the vacuum pump still turned on, a weight of 12 kg was evenly placed on the mold to compress the specimen and force out any leftover excess water that might have been caught in the now-thickened slurry. The specimen was manually demolded onto a flat, rectangular surface after the vacuum pump had been running for another 2–3 min. The demolded specimens were then left in the laboratory’s air for 15–20 min before being placed in a high-humidity chamber to undergo the cement hydration and/or curing process. The fiber cement board specimens made in this way were placed in a high humidity chamber set at 25 ± 2 °C and 95% relative humidity to allow them to go through the cement hydration process and/or curing.

Following 7 and 14 days of hydration, the cured fiber cement board specimens were tested for flexural strength and flexural behavior (toughness) in compliance with BS EN 12467 [35] specifications. A total of 10 specimens were made for each mix design; 5 were tested after a period of 7 days, and 5 more after 14 days of curing. The thickness of each specimen ranged from 8 to 10 mm. The mold set-up for the lab-simulated Hatschek process and a selection of the FCB specimens produced using the laboratory-based Hatschek technique are displayed in Figure 3.

## 3. Characterization of the Manufactured Cement Composite Boards

### 3.1. Mechanical Property Test

**Figure 4:**
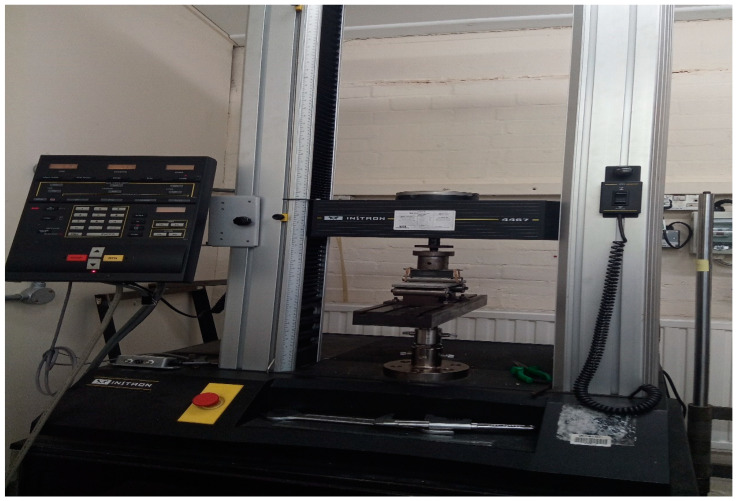
Bending test of specimen in the lab using the Instron 4467 Electromechanical Testing Machine, Norwood USA.

The cured specimens undergo a three-point bending/flexural test utilizing the Instron 4467 Electromechanical testing equipment as per BS EN 12467 (2012) specifications. The specimen must be simply supported so that one support is fixed and the other is free to move to align the specimen, following the standard. Additionally, each support’s upper face must be rounded to a radius of more than 3 mm but less than 25 mm to comply with the requirement outlined in the BS EN 12467 (2012) standards. Figure 4 shows the testing machine applying the load on the sample. To make sure that breakage occurred within 10 to 30 s of loading, each specimen was tested in bending at a constant rate of loading with a crosshead speed of 5 mm/min. This ensured that the specimen satisfied the requirements of the BS EN 12467 (2012) standards. Throughout the test, the supports’ span was adjusted and maintained at 125 mm. The modulus of rupture (*MOR*), as defined by the standard, is the flexural strength of the fiber cement board and is determined by applying the Equation (1) formulation.

(1) $$MOR=\frac{3FL_{s}}{2be^{2}}$$

Here,

*F* is the breaking load (N);

*L*_s_ is the span between the axes of the supports (mm);

*B* is the width of the specimen (mm);

*e* is the thickness of the specimen (mm).

### 3.2. Determination of Moisture Movement of Bamboo-Fiber-Reinforced Composite Boards

To have an insight into the characteristic behavior of the cement board when exposed to different weather conditions, a moisture movement test was conducted in a Gallenkamp TH340L environmental conditioning chamber capable of conditioning specimens at temperatures of −40 °C to 180 °C and at a relative humidity of 10% to 98% following ASTM C1185-08 (2016) [36]. Three (3) specimens were prepared for testing. Sample dimensions were 80 mm wide and 100 mm long. The specimens were conditioned to practical equilibrium at a temperature of 23 ± 2 °C and relative humidity of 30 ± 2%. After conditioning, each specimen was measured for length to the nearest 0.02 mm. The specimens were further conditioned to practical equilibrium at a temperature of 23 ± 2 °C and relative humidity of 90 ± 5%. Each specimen’s length was once again measured and recorded. These values were used to calculate the moisture movement (% change in length) using Equation (2), as follows.

(2) $$L_{m}=\frac{L_{90RH}-L_{30RH}}{L_{30RH}}\times 100$$

Here,

*L*_m_ is the linear moisture movement or the change in length (%);

*L*_90RH_ is the length of the specimen at RH of 90%, (mm);

*L*_30RH_ is the length of the specimen at RH of 30%, (mm).

### 3.3. Water Absorption Characteristics of Bamboo-Fiber-Reinforced Composite Boards

Water absorption was conducted following ASTM C1185-08 (2016) [36]. Three (3) specimens of each composition of the boards, measuring 180 mm by 80 mm, were dried to constant weight in an oven at a temperature of 90 ± 2 °C. After drying, the specimens were cooled in a desiccator and thereafter weighed to the nearest 0.01 g. The specimens were then immersed in clean water at 23 ± 2 °C for 48 ± 8 h. At the end of this period, each specimen was carefully blotted dry and then weighed again. The percentage of water absorption was calculated using Equation (3).

(3) $$W_{A}=\frac{W_{S}-W_{D}}{W_{D}}\times 100$$

Here,

*W*_A_ is the water absorption, (%);

*W*_D_ is the dry weight of the specimen, (g);

*W*_S_ is the saturated weight of the specimen, (g).

### 3.4. Determination of the Density of Manufactured Specimens

Following the Archimedes principles and using the water displacement method, the density of the manufactured cement boards was calculated in compliance with ASTM C1185-08 (2016) [36]. Three (3) specimens of each composition of the boards, measuring 180 mm by 80 mm, were first dried to constant weight in an oven at a temperature of 90 ± 2 °C. After drying, the specimens were cooled in a desiccator and thereafter weighed to the nearest 0.01 g. The specimens were then submerged in clean water at 23 ± 2 °C for 48 ± 8 hrs. At the end of this period, each specimen was weighed underwater to obtain the suspended weight using the Archimedes principles. Thereafter, the specimens were carefully blotted dry and the saturated weight was measured and recorded. The density of the specimens was calculated following Equation (4) as shown and described below.

(4) $$D=\frac{W_{D}\;}{W_{S}-W_{w}}\times \rho w$$

Here,

*D* = Density, (g/cm^3^);

*W*_D_ = Dry weight of specimen, (g);

*W*_S_ = Saturated weight of specimen, (g);

*W*_w_ = Weight underwater (suspended weight), (g);

ρ_w_ = Density of water, (g/cm^3^).

### 3.5. Morphological Examination of the Fractured Specimens Using SEM

Using a scanning electron microscope (SEM), TESCAN VEGA 3, with an Oxford instrument detector, sourced from TESCAN Worldwide, Brno., Czech Republic, the fractured surfaces of the fiber cement board specimens were examined for fiber failure/fracture mechanisms as well as fiber dispersion within the composites. To mitigate specimen charge and enhance conductivity for improved imaging, all fractured specimens were examined in the low-vacuum mode.

## 4. Results and Discussion

### 4.1. TGA Thermograms of Bamboo Fiber

**Figure 5:**
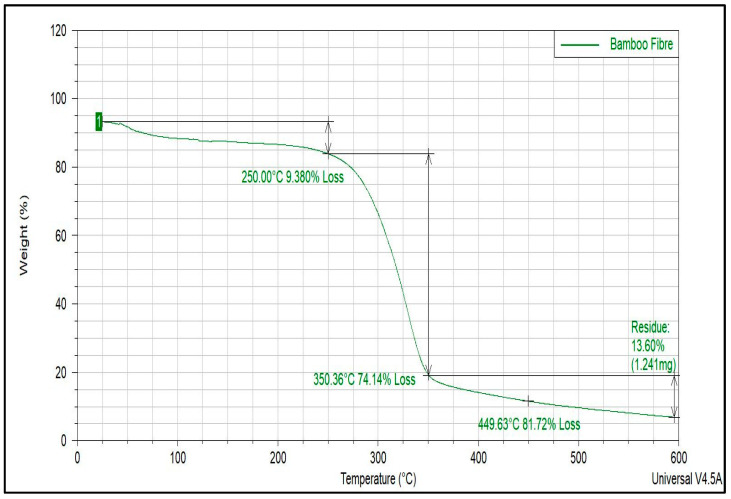
TGA curves of bamboo fiber in a nitrogen atmosphere.

The thermogravimetric properties of bamboo fiber are depicted in Figure 5. It was observed that the thermal properties of the fiber followed a three-stage degradation profile. The first stage of the degradation profile occurred over a temperature range of 25–250 °C; this stage represented the removal of the moisture contents from the fiber. The second stage of the degradation profile represented the decomposition of the non-crystalline constituents of the fiber (i.e., lignin and hemicellulose) because they were less thermally stable. Furthermore, it was observed that at a temperature of about 350 °C, the bamboo fiber had lost approximately 74% of its weight; this was because bamboo fibers contain a higher percentage of lignin and hemicellulose than most other vegetable fibers. This was confirmed in the report by Pacheco-Torgal et al. [1] when they reviewed vegetable fibers used as reinforcements in cementitious building materials; they concluded, in their research, that bamboo fibers generally contain more lignin and hemicellulose than is found in other vegetable fibers. Additionally, at a temperature of 350 °C, which represented the beginning of the third stage of the degradation profile, the crystalline constituents of the fiber had not decomposed. This shows that the fiber will not degrade during the cement hydration process because the hydration temperature is only about 100 °C; hence, the degradation of the fiber during the cement hydration process will not occur. Furthermore, the TGA curve revealed that at a temperature of 600 °C, the residual weight of the bamboo fiber was only 13.60%. The lower fraction of the char content left after the test further proved that the fiber was thermally stable; this meant that a smaller amount of bamboo fiber was consumed during the test. This agreed with the findings of Roma et al. (2008) [37], which reported that the smaller the amount of char content remaining at the end of the test was, the better the thermal properties of the material were. Hence, the bamboo fiber showed a higher resistance to thermal degradation.

During the water vacuuming stage of the Hatschek process, the kraft pulp fiber is employed to create a network that holds the cement particles in place during the industrial manufacturing of fiber cement boards [29,38,39]. Using the kraft pulp as both a processing fiber as well as a reinforcing fiber in the lab-simulated Hatschek process offers numerous advantages such as the fiber’s increased surface roughness, which boosts the fiber’s ability to adhere to the matrix. An additional benefit is that these fibers’ short length helps with the homogeneity and distribution of other reinforcing natural fibers within the matrix, hence strengthening the fiber–matrix link and, as a result, the effectiveness of reinforcement.

**Figure 6:**
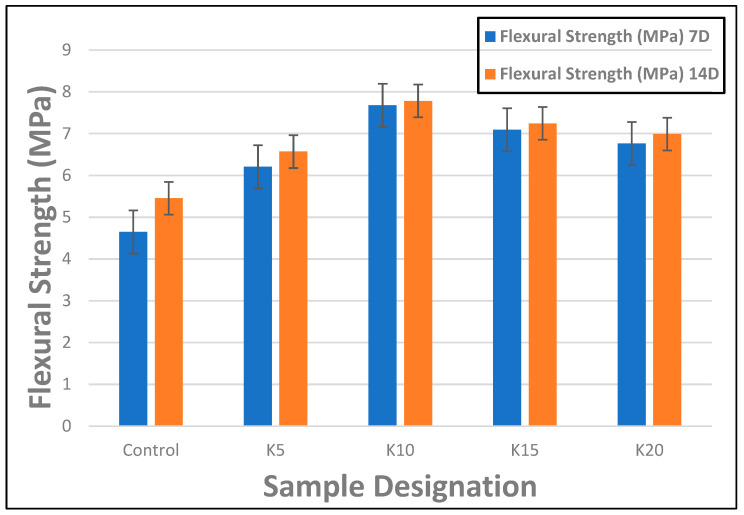
Flexural strength of FCBs reinforced with 5–20 wt.% of kraft fiber (7 and 14 days) (the ‘antennae’ indicate a standard error).

The flexural strengths of FCBs produced with varying percentages of kraft pulp at 7 and 14 days of hydration are shown in Figure 6. It was observed that the flexural strength of the composite board increased steadily as the period of hydration increased from 7 to 14 days due to the continuous hydration process, which produced cement hydration products such as calcium silicate hydrate (C-S-H) and Ca(OH)_2_. Furthermore, the flexural strength of the composite board increased steadily and consistently as the kraft pulp reinforcement content increased up to a maximum of 10 wt.%. After this point, there was a gradual drop in the flexural strength of the composite board, which was believed to be caused by the presence of excess kraft fibers in the composite that did not have cement particles to react with and hence constituted a weak point within the composite, leading to the observed reduction in flexural strength. In accordance with the research by Khorami and Ganjian [40], there should be a balance between the percentage of fibers and the cementitious matrix particles in the composite. In their research on the effect of fiber content on the flexural behavior of cement composite reinforced by waste kraft pulp, the authors examined the influence of varying residual kraft pulp contents (up to 14%) on cementitious composites’ flexural strength. They concluded that the ideal reinforcement threshold was 8–10% by mass of the cement matrix. Additionally, it was found that the composite sample K5, which had 5% kraft pulp fiber reinforcement, had 34% and 37% increases in strength compared to the control sample, which had values of 4.64 MPa and 5.45 MPa at 7 and 14 days, respectively for flexural strength. In a similar vein, the composite sample K10, which contained 10% kraft pulp fiber reinforcement, showed 65% and 43% increases in flexural strength over the control sample at 7 and 14 days, with flexural strength values of 7.68 MPa and 7.78 MPa, respectively. This demonstrated that the FCB can achieve optimal flexural strength by adding kraft pulp fibers up to a maximum of 10% by weight of the cementitious matrix.

**Figure 7:**
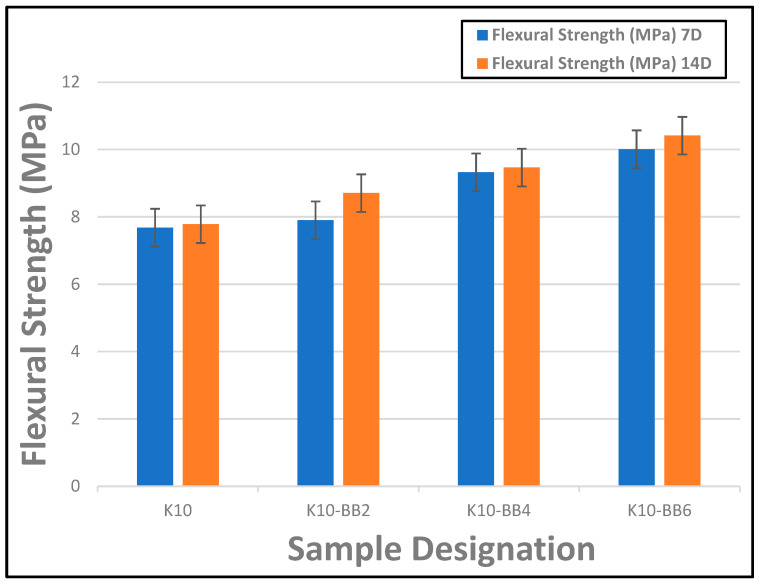
Flexural strength of FCBs reinforced by 10 wt.% of kraft pulp and 2–6 wt.% of bamboo fiber (7 and 14 days) (the ‘antennae’ indicate a standard error).

The flexural strength of composite board reinforced with kraft pulp and 2–6 wt.% of bamboo fibers is shown in Figure 7. It was observed that the presence of the bamboo fibers as reinforcements in the cement composite boards produces a gradual improvement in the flexural strength of the board, although at a lower reinforcement percentage, the effect of the fiber addition was very small, with only a 10% improvement in strength recorded. However, an increase in the percentage of fiber reinforcements led to a further improvement in the flexural strength of the composite boards, producing an optimum result of 10.41 MPa at 6 wt.% fiber loading, leading to a 34% overall improvement in flexural strength compared to the sample without fiber addition (reference sample K10). This maximum improvement in flexural strength of the board at 6 wt.% fiber addition could be traced to the satisfactory interaction at the fiber–matrix interface as well as to the uniform fiber distribution within the composite boards. A similar observation was noted in the work of Correia et al. [29] when they reinforced Ordinary Portland Cement with bamboo organosolv pulp at a varying percentage of 6–12%. They reported that the mechanical properties of the composites containing 6% bamboo organosolv pulp displayed the best properties before cracking. Furthermore, they concluded in their findings that the composites reinforced with more than 6% bamboo organosolv pulp suffered lower mechanical performance due to the excessive porosity imposed by the added fiber; however, the toughness of these composites in the post-cracking conditions was higher. Therefore, the current research suggests that depending on the intended area of application of the composite, an optimum percentage of reinforcement should be selected. That is, the application determines whether a material must have more mechanical strength or greater impact resistance.

**Figure 8:**
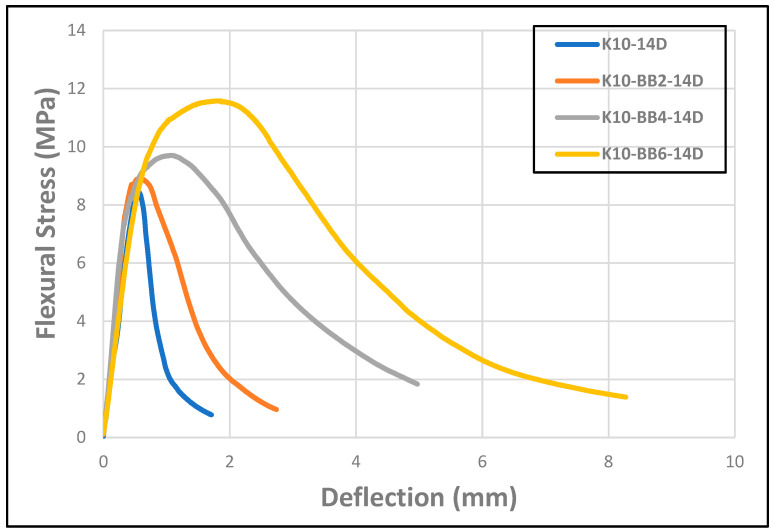
Flexural behavior of FCBs reinforced by 10% of kraft pulp and 2, 4, and 6 wt.% of bamboo fiber (14 days).

Figure 8 shows the flexural responses of the cement board reinforced with bamboo fibers after 14 days of curing in the high-humidity chamber. The curves display the mechanical behavior of the cement composite boards containing different percentages of bamboo fibers. It was observed that the reference sample K10, which had no bamboo fiber addition, showed both the minimum flexural stress as well as the least response to deflection. However, all the composites with bamboo fiber reinforcement showed better flexural stress and improved responses to deflection, credited to the presence of the bamboo fiber reinforcement. Furthermore, it was observed that the area under the stress-deflection curves (tenacity) for composites containing bamboo fiber addition was significantly greater, particularly for composite boards reinforced with 4 and 6 wt.% bamboo fiber. This shows that the addition of the bamboo fiber improves the strain-hardening behavior of the composite boards, thereby allowing a continuous increase in the stress values even after the initiation of the first cracks within the composite board. This characteristic behavior reveals that the bamboo fiber fulfils a very important role as a reinforcing and toughening agent in the mechanical behavior of cement composite boards. Furthermore, a closer observation of the different curves in Figure 8 reveals that the composite sample K10-BB6, containing 6 wt.% of bamboo fiber as a reinforcement, shows the optimum flexural stress and the highest response to deflection, peaking at about 2 mm deflection compared to the reference sample K10, which had the least deflection response, peaking at approximately 0.6 mm. This optimum response to deflection behavior displayed by the composite sample K10-BB6 could be traced to the presence of the bamboo fibers that offered significant internal strength to the composite, enabling the smooth transfer of load from the brittle matrix to the fibers; hence, the composite did not fail catastrophically but showed a ductile-like transition to failure mode.

### 4.2. Effect of Varying Fiber Content on the Moisture Movement Characteristics of FCBs

**Figure 9:**
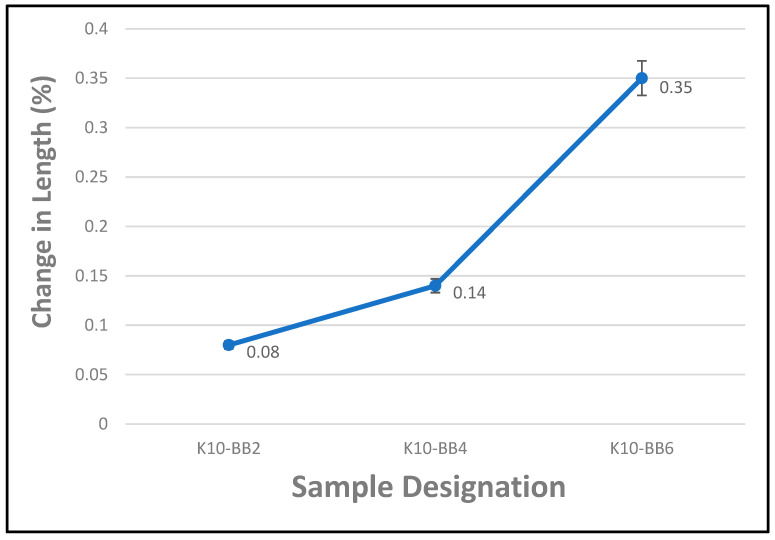
Percentage change in length of composite boards exposed to moisture movement (the ‘antennae’ indicate a standard error).

Cement composite boards reinforced with natural cellulose fibers may tend to absorb moisture when there is an increase in the relative humidity of their surrounding environment. This may lead to changes in the dimensional stability of the composite boards, and hence, swelling may occur. However, when the relative humidity of their environment drops, the composite boards may desorb moisture, which might result in a commensurate shrinking. However, this depends largely on the time of exposure and the various materials that constitute the composite board. This is because different materials contained in the cement board will absorb moisture at different rates due to their microstructural differences. Figure 9 shows the results of the percentage change in length of FCBs reinforced with varying percentages (2–6 wt.%) of bamboo fiber exposed to moisture movement over a period of 24 h. It was observed that an increase in the fiber content produced a corresponding increase in the moisture movement in the cement composite boards; this could be attributed to the hydrophilic characteristic nature of natural fibers. Furthermore, it was observed that the percentage change in length of the composite board also increased with an increase in fiber content; this could be attributed to the higher percentage of fiber loading, which may have attracted more moisture compared to the composite board with a smaller amount of fiber loading. The difference between the percentage change in length for composite boards with 2 wt.% and 6 wt.% fiber loading was about 0.27%. Even though it was noted that the fiber cement board was extremely pervious, water could easily penetrate it from any direction. It should be noted that the presence of water vapor or moisture alone will not cause damage to the composite board since the pores of a hygroscopic material like a fiber cement board cannot be filled with water absorbed from vapor or moisture in the air. Furthermore, according to the research authored by Cooke and by Tonoli et al. [41,42], the equilibrium water content as a percentage of the oven dry weight at normal air humidity and temperature is about 7% compared to the approximately 35% needed to completely saturate the cement board. As a result, the pores of the fiber cement board have enough space for the moisture to spread without causing any damage to the composite boards. This assertion was further confirmed in another study by Maan [43], in which they studied the moisture sensitivity and dimensional stability of carbonated fiber–cement composites.

### 4.3. Effect of Fiber Content on the Water Absorption Properties of Cement Composite Boards

**Figure 10:**
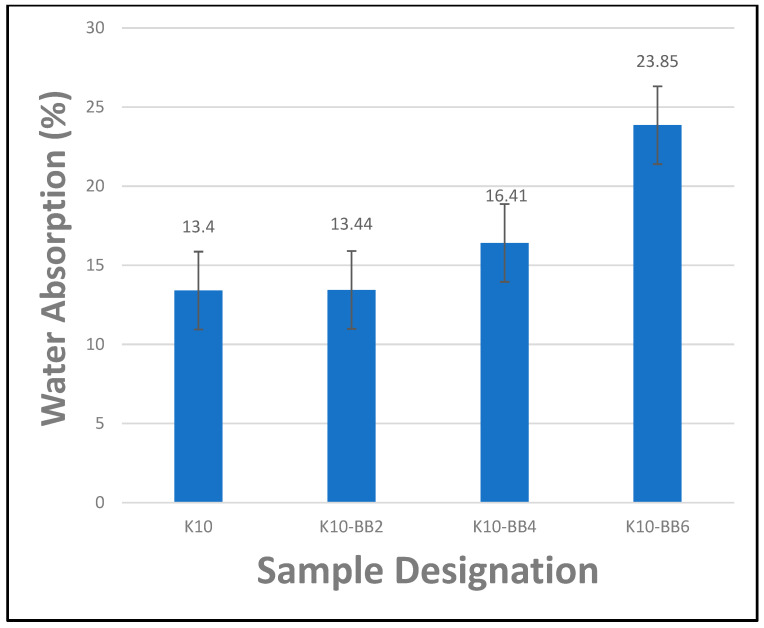
Percentage of water absorption of FCBs submerged in water for 48 h (the ‘antennae’ indicate a standard error).

The water absorption characteristics of cement composite boards reinforced with kraft pulp and bamboo fibers are shown in Figure 10. It is crucial to note that the various constituents of the composite boards will absorb water at different rates owing to the differences in their structural integrity. In the present study, it was believed that the bamboo fiber constituent of the composite board would absorb water much faster than the cement paste particles due to their characteristic nature, as mentioned previously in the report, particularly during the first few hours of immersion in water. However, it is quite difficult to estimate when the fiber would have attained its water saturation point. It is believed that this would take much longer for the cement paste particles. Hence, the water transport mechanism within the composite board is expected to move from the fiber and matrix to the interfacial zones between the constituents of the composite. This means that the fiber–matrix interfaces within the composite board are not immediately disturbed by water molecules until the reinforcing fiber and the matrix have almost reached their respective water saturation points. Therefore, it was observed, from the current study, that the reference sample (K10) containing kraft pulp fiber only absorbed the least volume of water, with a value of 13.4%, when compared to the other composite boards containing bamboo fiber reinforcements. Furthermore, it was also observed that increasing the bamboo fiber reinforcement contents from 2–6 wt.% led to a corresponding increase in the water absorption property of the composite board. This characteristic behavior is attributed to the inherent nature of the bamboo fiber being hydrophilic (i.e., having a strong affinity for water). Hence, the composite board K10-BB6, containing a higher percentage of bamboo fiber loading, absorbed the highest volume of water, with a value of 23.85%.

### 4.4. Effect of Fiber Content on the Density of Cement Composite Boards

**Figure 11:**
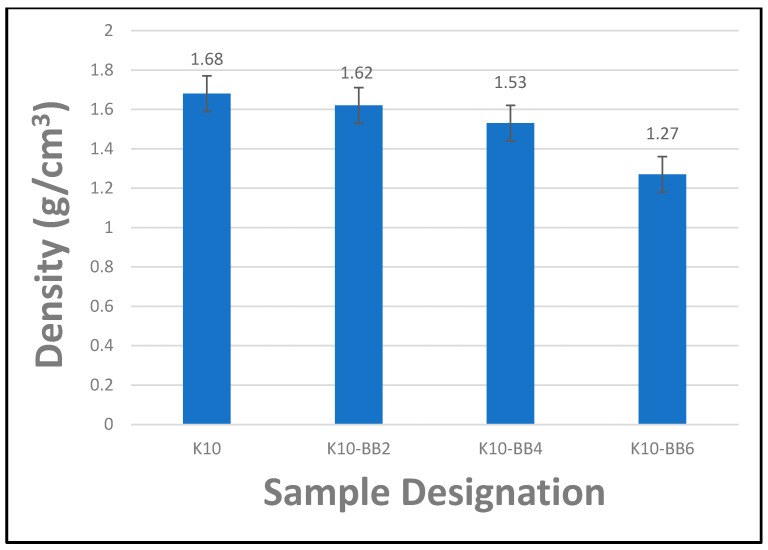
The density of FCBs reinforced with kraft pulp and bamboo fibers (the ‘antennae’ indicate a standard error).

Figure 11 presents the results of the relationship between the density of composite boards and the amount of fiber reinforcement. It can be noted from the graph that all the composite boards containing bamboo fiber reinforcement had a lower density compared to the reference sample K10, which had only kraft pulp fiber as reinforcement. Furthermore, it was observed that as the percentage of bamboo fiber reinforcement increased from 2 to 6 wt.%, the density of the corresponding composite boards reduced gradually; this was attributed to the lightweight characteristics of the bamboo fiber. Hence, all the composite boards reinforced with bamboo fiber have densities with respective values of 1.62, 1.52, and 1.27, presenting 4%, 10%, and 32% reductions in density, respectively compared to the reference sample without bamboo fiber reinforcement. This shows that using bamboo fiber as a reinforcement in fiber cement composite boards could provide lighter boards for building applications. This would result in saving costs in terms of transporting materials and the installation of the final products. The density values recorded for the bamboo-fiber-reinforced composite boards in the current research study are very competitive with values reported by other researchers in the literature [43,44,45].

### 4.5. SEM Micrographs of FCBs Reinforced with 10 wt.% of Kraft Pulp and 4–6 wt.% of Bamboo Fibers

**Figure 12:**
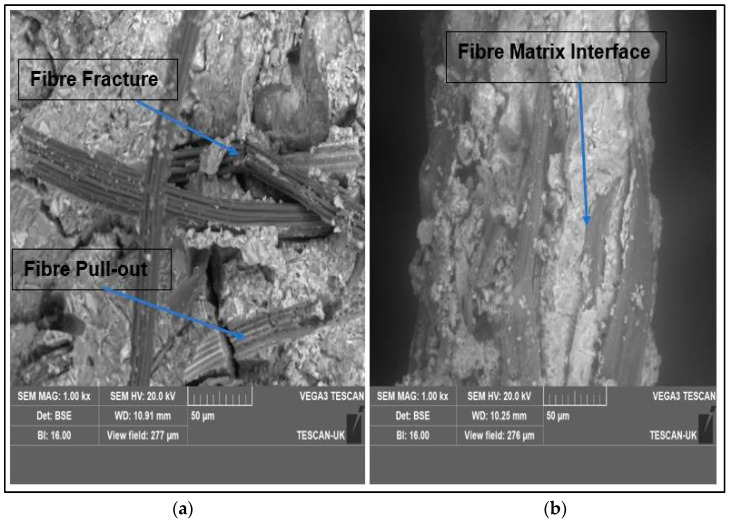
SEM micrographs of fractured surfaces of samples (**a**) K10-BB4 and (**b**) K10-BB6.

Backscattered scanning electron microscope images of the fractured surfaces of kraft-pulp- and bamboo-fiber-reinforced composite boards are depicted in Figure 12. The SEM micrograph depicted in Figure 12a reveals that the composite failed by a combination of fiber fracture and fiber pull-out as annotated on the image. Furthermore, good interfacial bonding between the fiber and the cement matrix was also observed in the micrograph depicted in Figure 12b. Perhaps this could be the reason for the optimum strength displayed by the composite sample K10-BB6. Additionally, in both composite samples shown in Figure 12a,b, there was no evidence of the migration of cement hydration products to the lumen of the fiber. This can only suggest that the fiber remains unharmed within the composite board despite its interactions with the high-alkali environment of the cement matrix. This contrasts with the opinion raised in the work of Ardanuy et al. [46] when they studied fiber–matrix interactions in cement mortar reinforced with cellulosic fibers. The authors claimed that they suspected the presence of precipitated inorganic particles on the fiber’s surface and/or lumen. They further argued that the fibers were saturated with the precipitation compounds of the cement matrix, primarily in the zones nearer to the fiber–matrix interface. However, the different behavior observed in the present research can be justified, possibly due to the level of purity and/or hornification of the fiber studied.

## 5. Conclusions

The present study assessed the mechanical and durability performance of kraft-pulp-and bamboo-fiber-reinforced cement composite boards. After conducting various tests on the developed cement boards, the following conclusions could be drawn.

- The TGA study of the fiber revealed that bamboo fiber is thermally stable and will not degrade during the cement hydration process.
- To achieve optimum flexural strength and toughness in the composite board, a maximum composition of 10 wt.% kraft pulp and 6 wt.% bamboo fiber should be employed.
- The durability study revealed that lightweight structural materials for interior building applications can be produced from the combination of kraft pulp and bamboo fibers.
- SEM morphology of the composite revealed good interaction at the fiber–matrix interface. The composite displayed a combination of fiber fracture and pull-out as the mode of failure.
- In conclusion, the bamboo-fiber-reinforced composite boards developed in this study presented flexural strength and toughness characteristics that are acceptable for fiber cement flat sheets according to relevant standards. Hence, the composite boards developed from this research may be recommended for internal building applications in low-cost housing estates in developing countries.

## Figures and Tables

**Table 1:** Mechanical and physical characteristics of the bamboo fiber.

Fiber Type	Tensile Strength (MPa)	Young’s Modulus (GPa)	Elongation at Break (%)	Average Length (mm) ^a^*	Average Diameter (mm) ^b^*	Aspect Ratio (Length/Diameter)
**Bamboo**	401	23 ± 3.59	0.16 ± 0.07	5.3	0.03	176.67

a*, b*—Average of 10 measurements obtained from high-resolution Nikon Eclipse ME600 Microscope.

**Table 2:** The mix design for composite production.

Mix	Sample Designation	Matrix Material (Cement) (g)	Kraft Pulp Fiber (K) (g)	Bamboo Fiber (BB) (g)
Stage 1 (Cement and kraft pulp fiber)				
1	Control	200	0	0
2	K5	190	10	0
3	K10	180	20	0
4	K15	170	30	0
5	K20	160	40	0
Stage 2 (Cement, kraft pulp, and bamboo fiber)				
6	K10-BB2	176	20	4
7	K10-BB4	172	20	8
8	K10-BB6	168	20	12

## Data Availability

Data are contained within the article.
